# BDHusk: A comprehensive dataset of different husk species images as a component of cattle feed from different regions of Bangladesh

**DOI:** 10.1016/j.dib.2023.110018

**Published:** 2023-12-28

**Authors:** Ifteasam Islam Jahin, Munni Khatun, Md. Tarequl Islam, Md. Wahidur Rahman, Ishrat Zahan Raka

**Affiliations:** a Department of Computer Science and Engineering, Khwaja Yunus Ali University, Sirajganj, Bangladesh; b Department of Computer Science and Engineering, Mawlana Bhasani Science & Technology University, Tangail, Bangladesh; c Department of Computer Science and Engineering, Uttara University, Dhaka, Bangladesh

**Keywords:** Cattle feed, Computer vision, Deep learning, Image classification, Machine learning, Agricultural development

## Abstract

This study presents a recently compiled dataset called “BDHusk,” which encompasses a wide range of husk images representing eight different husk species as a component of cattle feed sourced from different locales in Sirajganj, Bangladesh. The following are eight husk species: Oryza sativa, Zea mays, Triticum aestivum, Cicer arietinum, Lens culinaris, Glycine max, Lathyrus sativus, and Pisum sativum var. arvense L. Poiret. This dataset consists of a total of 2,400 original images and an additional 9,280 augmented images, all showcasing various husk species. Every single one of the original images was taken with the right backdrop and in enough amount of natural light. Every image was appropriately positioned into its respective subfolder, enabling a wide variety of machine learning and deep learning models to make the most effective use of the images. By utilizing this extensive dataset and employing various machine learning and deep learning techniques, researchers have the potential to achieve significant advancements in the fields of agriculture, food and nutrition science, environmental monitoring, and computer sciences. This dataset allows researchers to improve cattle feeding using data-driven methods. Researchers can improve cattle health and production by improving feed compositions. Furthermore, it not only presents potential for substantial advancements in these fields but also serves as a crucial resource for future research endeavors.

Specification Table

**Table:** 

Subject	Computer Sciences, Agricultural Sciences, Food and nutrition science
Specific subject area	Computer Vision classification of different types of Husks.
Data format	Raw, Analyzed, and Filtered.
Type of data	Image.
Data collection	From the range of two months in July to August 2023. We collect quality images of husks from various places within Sirajganj, Bangladesh. We used the camera of the Redmi Note 8 and the Samsung M21. Afterward, any kind of motion blur, noise, or low-resolution images were removed from the dataset. We have taken 2400 images of these eight species in different light conditions, e.g., under direct sunlight at different times of the day. The dataset also provides 9,280 augmented images. In the whole process of data collection, we have tried to capture the images as nearly natural as they look in natural light.
Data source location	This data has been collected from our local places. Such as: 1. Sirajganj Sadar (6700), Sirajganj. 2. Shahjadpur (6770), Sirajganj. 3. Belkuchi (6740), Sirajganj. 4. Enayetpur (6751), Sirajganj.
Data accessibility	Country: Bangladesh. Repository: Mendeley Data. DOI: 10.17632/h754ntdtfx.1 URl: https://data.mendeley.com/datasets/h754ntdtfx/1

## 1 Value of the Data

• The “BDHusk” dataset is valuable because it provides a huge collection of husk images from eight different types of husks in Bangladesh. This dataset can be valuable in various research fields, such as agriculture, animal science, and data analysis.
• BDHusk comprises 2400 original images and 9280 augmented images so that the researchers can effectively contribute to data analysis and compare the various types of husks.
• Researchers can use the dataset to assess the nutritional value of various husk types and their usefulness as a component of cattle feed. This knowledge can be used to create more economical and balanced cattle feeds.
• To evaluate the environmental effects of cattle farming, it can be essential to understand the composition of cattle feed, especially husk. Researchers can use the dataset to determine the carbon footprint and resource usage of various feed compositions.
• This dataset can be used to develop an application for the identification and classification of husks in the field of cattle feed.
• Furthermore, this dataset can contribute to sustainable agriculture by helping identify opportunities for recycling agricultural waste materials like husks into beneficial livestock feed, reducing waste, and promoting eco-friendly farming practices.

## 2 Objective

The primary objective of establishing the “BDHusk” dataset is to provide an exhaustive and well-structured resource that supports researchers, particularly those in the fields of machine learning and deep learning, in advancing the optimization of cattle feed formulations. This dataset intends to give useful insights and make it easier to construct predictive models and algorithms that improve the efficacy of cattle feed and the nutritional content it provides by utilizing husk as an essential component. The dataset includes eight different types of husk images, namely Oryza sativa, Zea mays, Triticum aestivum, Cicer arietinum, Lens culinaris, Glycine max, Lathyrus sativus, and Pisum sativum var. arvense L. Poiret. This dataset serves as a foundation, providing a wealth of data for the development of predictive models and algorithms that can considerably improve the efficacy, cost-effectiveness, and nutritional value of cattle feed. Essentially, BDHusk empowers researchers to advance the field of cattle nutrition by leveraging the power of data-driven approaches to optimize feed formulations and, thus, improve cattle health and productivity.

## 3 Data Description

**Fig. 1:**
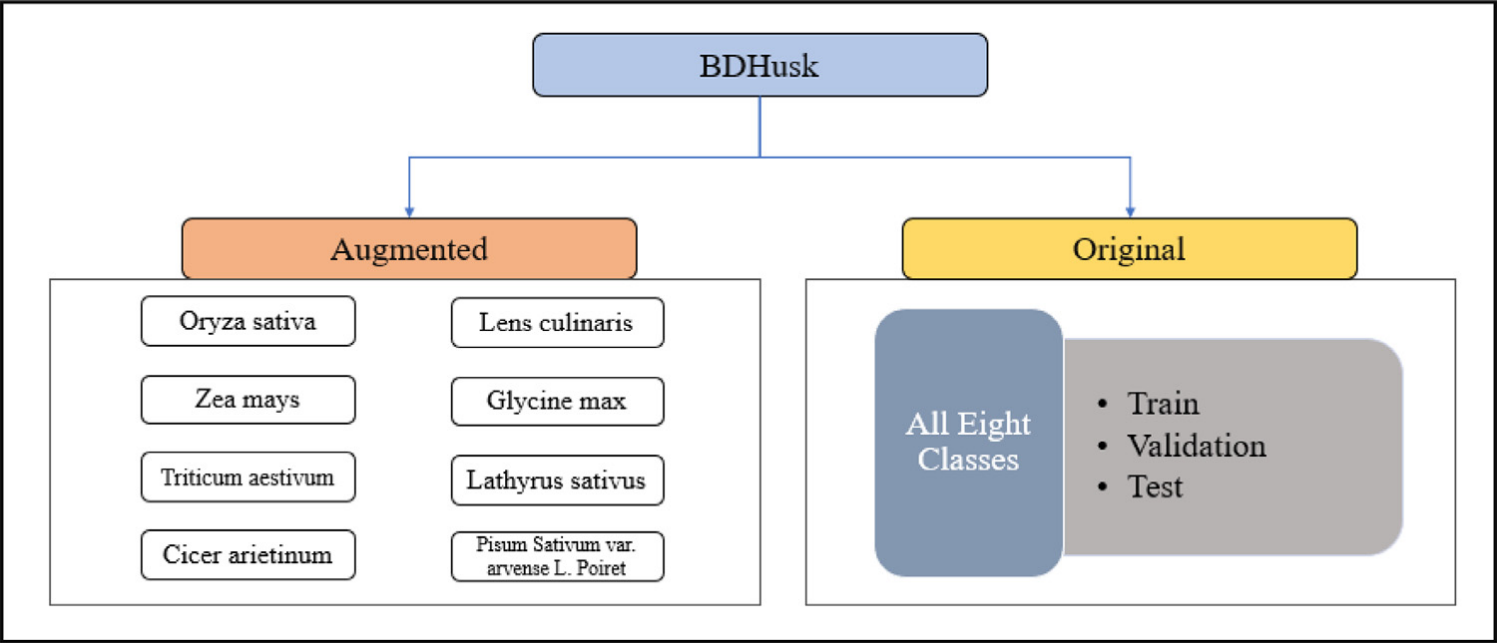
The directory structure of the BDHusk dataset proposal.

**Fig. 2:**
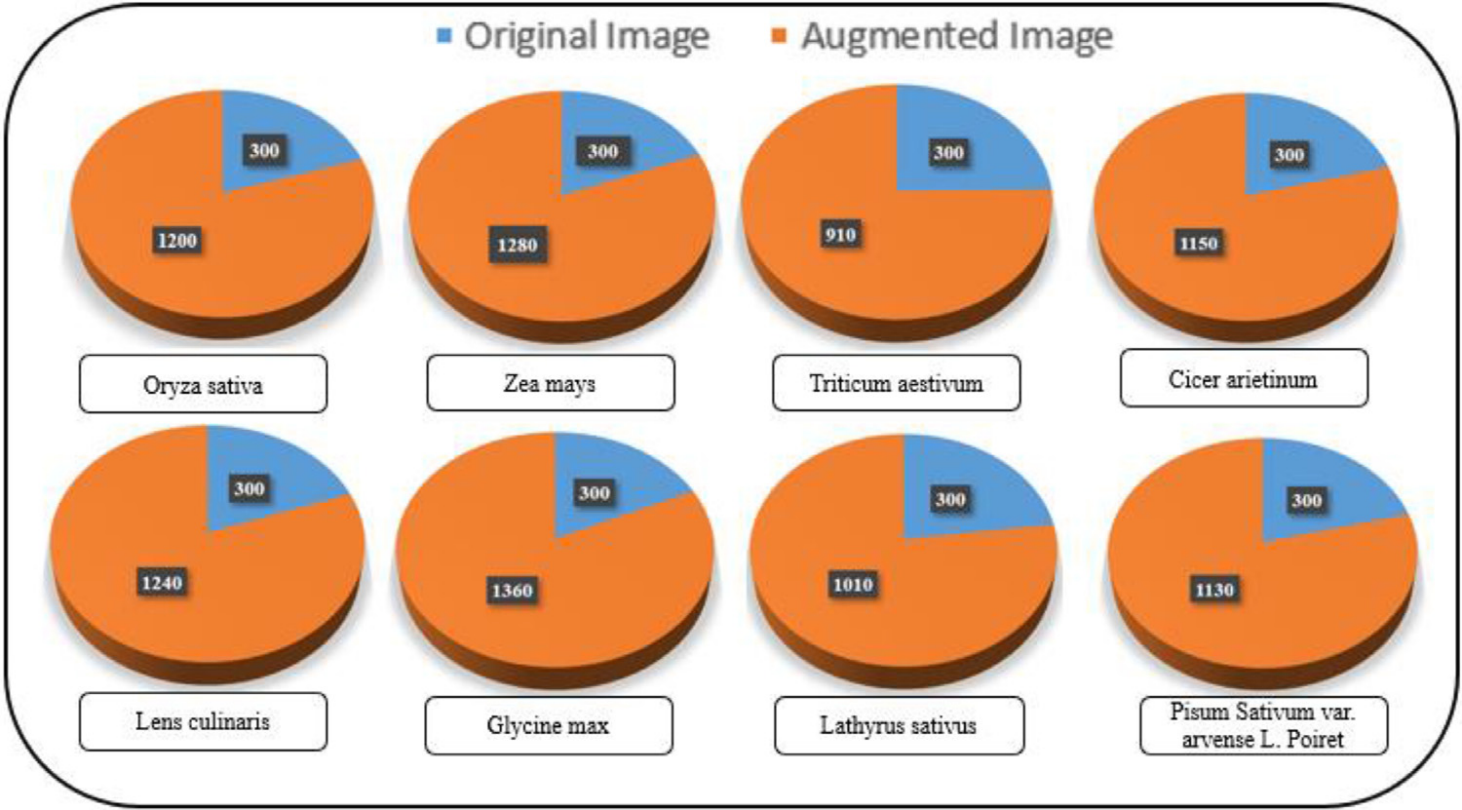
Image distribution based on husk species in the BDHusk dataset.

**Fig. 3:**
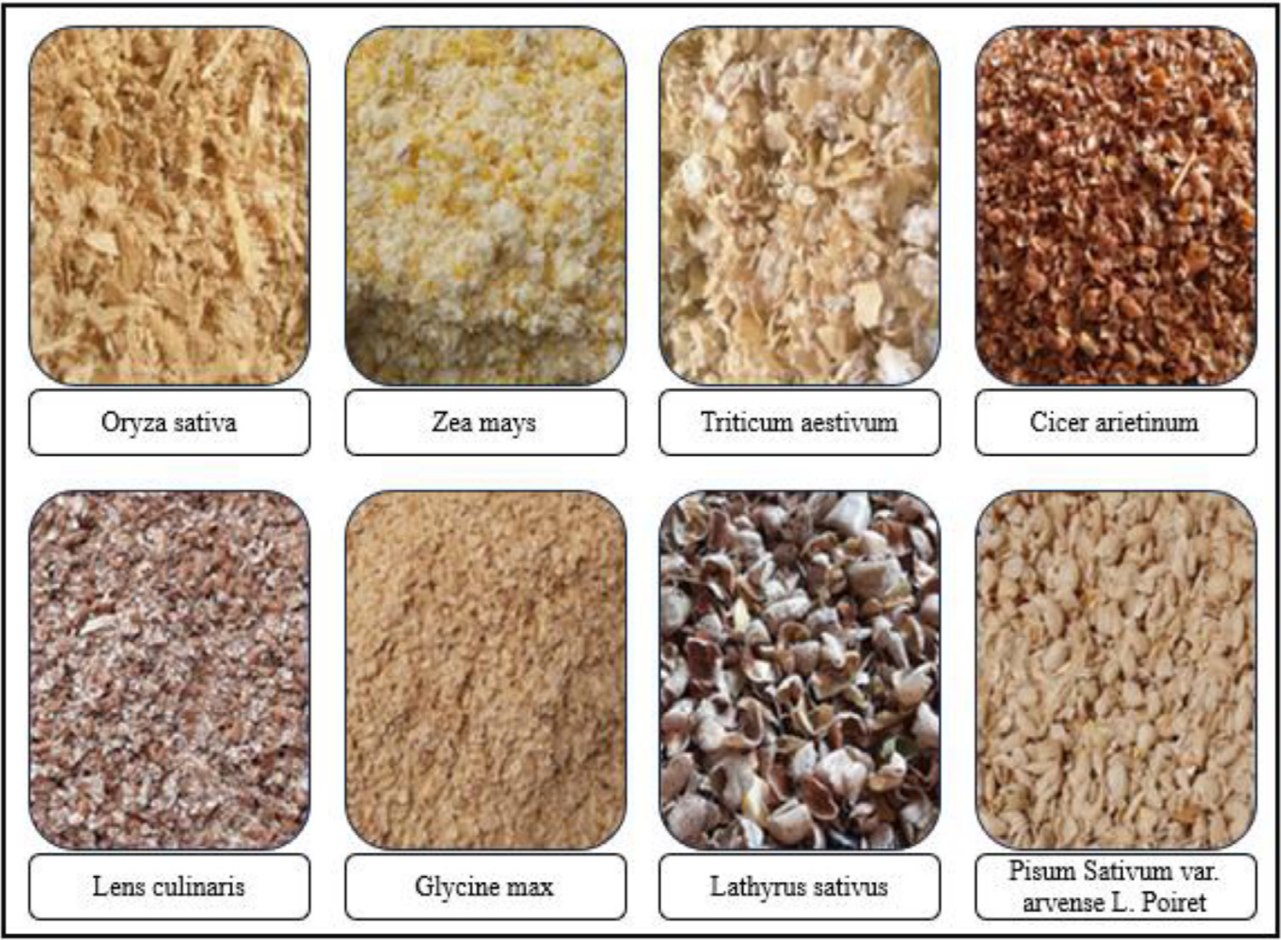
Sample images from each class of the BDHusk dataset.

**Fig. 4:**
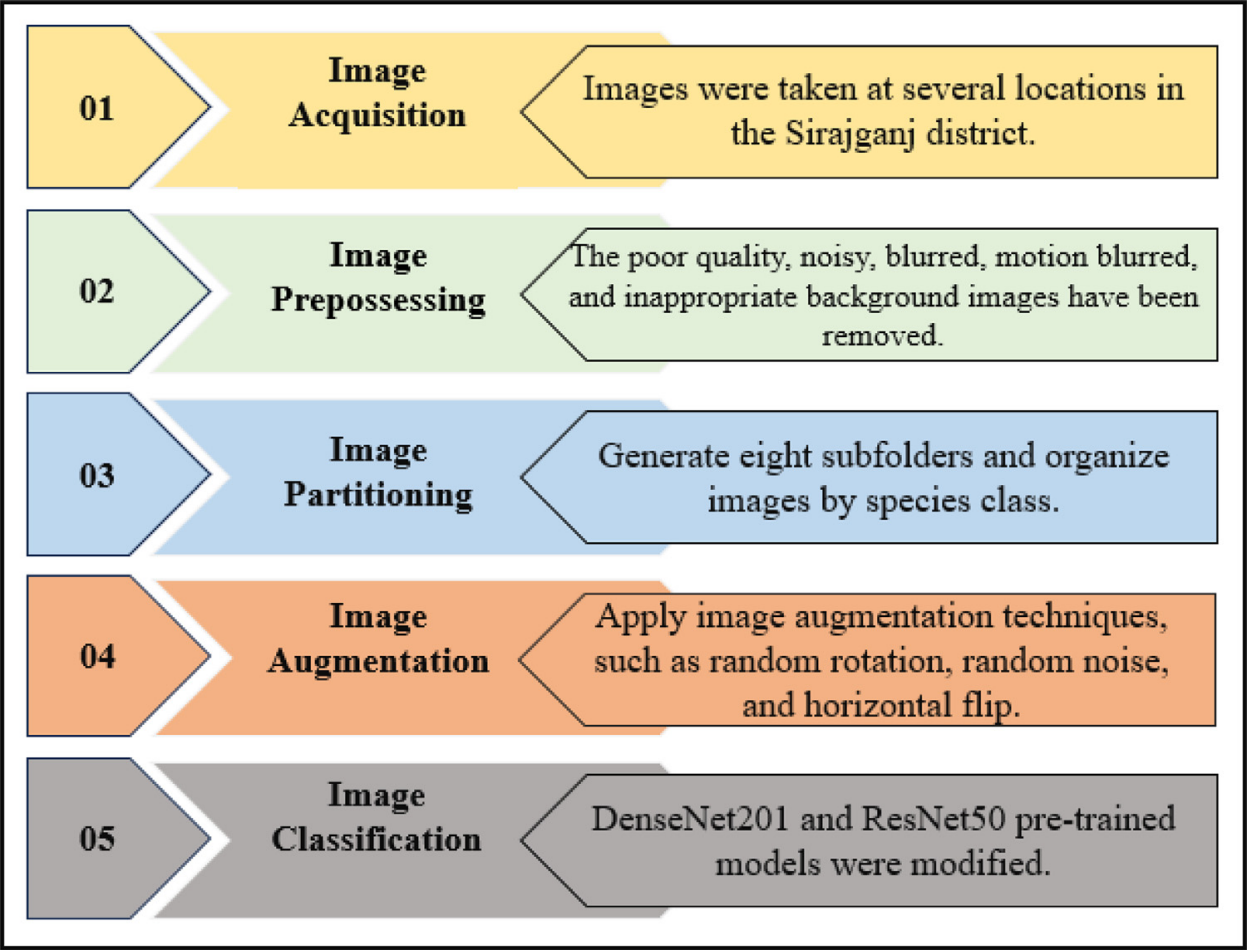
Processes involved in the development of the BDHusk dataset.

**Fig. 5:**
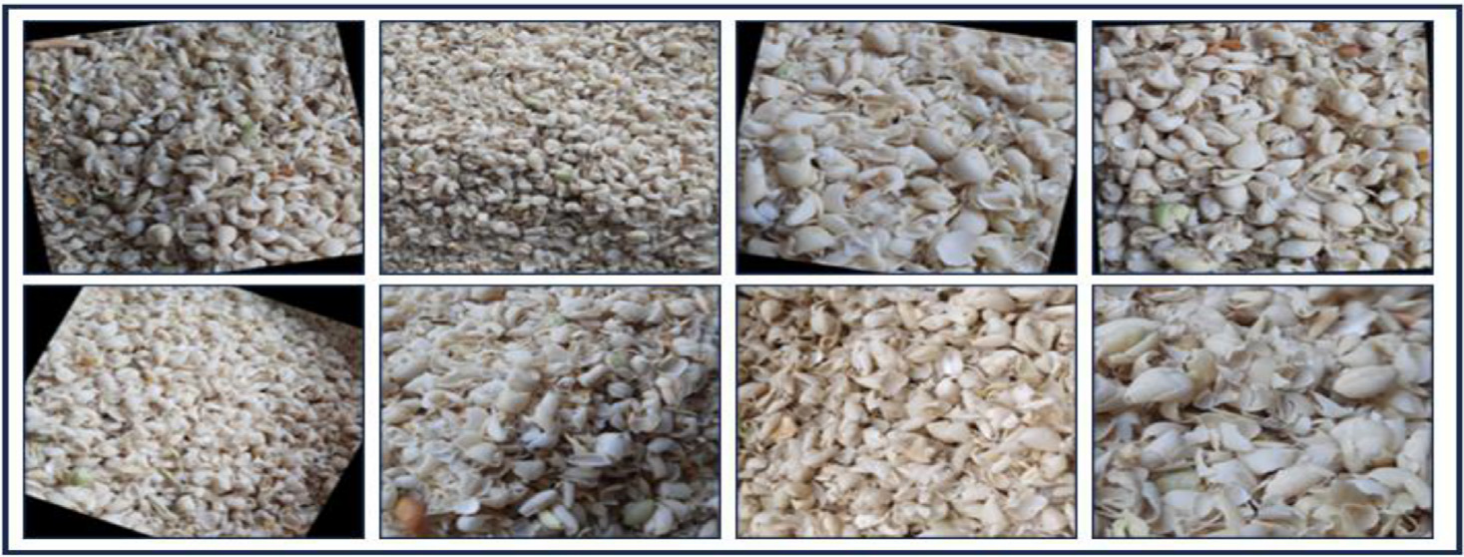
Some examples of Augmented Image from a class.

The “BDHusk” dataset consists of a compilation of images collected from multiple locations in Sirajganj, Bangladesh. The dataset comprises 2,400 original images and 9,280 augmented images, which are organized in the manner that is shown in Fig. 1. The main directory, referred to as “BDHusk”, consists of two subordinate directories. One directory is dedicated to storing the original husk images that were initially collected, while the other directory is designated for images that have undergone different image augmentation techniques. The first directory encompasses three distinct subdirectories, namely Train, Validation, and Test. Each of the three subdirectories contains a complete set of all eight classes of images. In the augmented directory, we offer class-specific augmented images for researchers to use based on their specific requirements (Fig. 2, Fig. 3, Fig. 4, Fig. 5).

Although there are many types of husks used as cattle feed, in the regions from which we collected data, eight types of husks are mainly used, and other types of husks are rarely seen outside of them. That is why these eight types of husks are used in our dataset. The following section provides an overview of the eight husk species that are encompassed within the “BDHusk” dataset, along with a short description of each:

### 3.1 Oryza sativa husk

Oryza sativa husk, commonly known as “Rich Husk”. The outermost coating of rice grains is called rice husk, and it is frequently seen as a waste product when rice is processed. It is a fibrous, light substance that separates from the rice kernel that is edible. The energy dispersive X-ray analysis conducted on the outer/inner surface of rice husk reveals the presence of various elemental constituents, these constituents, by weight, consist of carbon ranging from 9.55% to 18.74%, oxygen ranging from 30.90% to 35.51%, silica ranging from 58.19% to 43.17%, and potassium ranging from 1.36% to 1.66% [1]. Rice husk can be used to reduce waste treatment costs by removing heavy metals from wastewater and solving disposal issues, easy availability and low cost of rice husk in rice-producing regions are further benefits for its use [2].

### 3.2 Zea mays husk

Zea mays husk, widely recognized as “Corn Husk”. The term “corn husk” is used to describe the protective outer layer that covers the corn ear and it is composed of lignocellulosic fiber, which is known for its abundant cellulose content and minimal amounts of lignin and ash [3]. They are part of a balanced diet that is supplemented as necessary to satisfy the nutritional needs of cattle. Cattle must have sufficient access to water when fed corn husks or any other high-fiber feed to prevent dehydration and promote digestion.

### 3.3 Triticum aestivum husk

Triticum aestivum husk, usually known as “Wheat Husk”. Wheat husk, which is the top layer of wheat grains, is a good source of fiber that can be added to cattle feed. It gives cattle a source of roughage that helps their stomach and gut stay healthy. It is used as a feeding ingredient for cattle, and when appropriately incorporated into their diet, may provide several health and nutritional benefits. However, it's low in protein and certain essential nutrients, so it should be part of a balanced diet with proper supplementation to meet cattle's nutritional requirements. Wheat husks are abundantly generated each year, present a highly economical and eco-friendly option for producing carbonaceous materials, which makes wheat husk supercapacitors a highly valuable commodity in the rapidly growing power market [4].

### 3.4 Cicer arietinum husk

Cicer arietinum husk known as “Chickpea Husk”. Chickpea husk is an important part of cattle feed. It is the chickpeas' top shell that protects them. The chickpea husk is an incredibly significant pulse crop that effectively meets the protein requirements of livestock worldwide [5]. Chickpea husks are a notable dietary fiber source, as they contain up to 40% fiber in the form of celluloses, hemicelluloses and pectin [6]. Chickpea husk gives cattle important nutrients and helps their digestion when it is added to their food. It is good for cattle to eat because the fiber in it helps keep their rumens working well and their digestive systems healthy overall. It's a cost-effective and sustainable feed option, especially when fresh forage is limited. However, it should be balanced with other feeds to meet nutritional needs, and its composition can vary, so careful analysis is essential for optimal cattle nutrition.

### 3.5 Lens culinaris husk

Lens culinaris husk, usually known as Lentil husk. The outside layer of lentil seeds, called lentil husk, is a useful ingredient in cattle feed. Lentils are considered a nutrient-dense food legume due to their substantial energy value, essential minerals, complex carbohydrates (primarily slow-digestible starch and minimal unrefined fiber), and protein content [7]. It has a lot of fiber and protein, which makes it a great addition to livestock feed to make it healthier. Lentil husk helps cattle digest food properly and improves their gut health. It can help livestock receive nutrients better and generally be healthier if added to their food. It supports sustainable farming practices, offers roughage, and helps manage cattle weight. However, with the highest adsorption potential among all other agricultural wastes examined, lentil husk, an agricultural byproduct, was discovered to be a prospective low-cost adsorbent for lead removal [8].

### 3.6 Glycine max husk

Glycine max husk, widely recognized as “Soybean Husk”. Soybean husk, a highly valuable component extracted from the outer shell of soybeans, holds immense significance in the field of cattle nutrition due to a multitude of compelling factors. Soybean husk is a major byproduct of soybean oil manufacturing that is mostly used as livestock feed [9]. Soybean husk is a significant agricultural waste product that is made up of complex carbohydrates like 14–25% cellulose, 14–20% hemicellulose, 10–12% pectin, 7–10% uronic acid, 2–4% lignin, and 77–88% of the pectin as galacturonic acid [10]. Cattle require a high-protein diet for proper growth, muscular development, and general health, and soybean husk provides this. Soybean husk is an essential source of nourishment for cattle because it contains essential amino acids.

### 3.7 Lathyrus sativus husk

Lathyrus sativus husk, commonly known as “Grass Pea Husk”. It's also known as “Khesari Husk” [11]. This husk refers to the tough outer layer of the legume grass pea (Lathyrus sativus). The seeds inside the husk are what people eat, hence the husk must be removed during processing. These seeds are an economical food source with a significant protein content, making them a primary source of nutrition [11]. The fiber level of grass pea husk makes it a useful feed item for cattle, although it is not commonly received by humans. It's a common ingredient in animal feed because of the benefits it provides to cattle digestion as a source of nutritional roughage. The inexpensive and environmentally beneficial husk option is Lathyrus sativus husk [12].

### 3.8 Pisum Sativum var. arvense L. Poiret husk

Pisum Sativum var. arvense L. Poiret husk, popularly recognized as “Field Pea Husk”. Field pea husks are economically advantageous and nutritionally excellent sources of protein when ingested in conjunction with grains, which are characterized by a deficiency in key amino acids [13]. It refers to the outer covering of field peas, a variety of legume that is typically raised in agricultural contexts. This fiber, a natural byproduct of processing field peas, has multiple potential applications. It is high in dietary fiber, which can be advantageous for both animal feed and human food products. Field pea husk contains vital nutrients such as phosphorus, which promotes bone health and overall nutrition in cattle. Introducing field pea husk to cattle feed can improve growth, health, and performance in populations, making it a valuable addition to contemporary livestock nutrition strategies.

## 4 Experimental Design, Materials and Methods

The development process of the “BDHusk” dataset comprises five distinct steps. The sequential stages encompassed in the process include image acquisition, image preprocessing, image partitioning, image augmentation, and image classification. This section provides a concise overview of each of these procedures.

### 4.1 Image acquisition

The dataset covers eight unique categories of husks. The raw husk images were taken in different locations within Sirajganj District in Bangladesh, utilizing a Xiaomi Redmi Note 8 (with a 48 Megapixel camera, f/1.8, 26mm (wide), 0.8µm, PDAF, 8 MP, f/2.2, 120˚ (ultrawide), 1/4.0", 1.12µm) and Samsung M21(48MP f2.0 main camera with an 8MP f2.2 ultra-wide and 5MP depth sensor). All images were captured with a consistent natural daylight background. To optimize the appearance of husk features and reduce the presence of shadows, diffused lighting was utilized during the image acquisition process. We ensured that a consistent and authentic background was maintained throughout the capture process. To maintain a consistent level of image quality, we implemented regular checks throughout the acquisition process. The process entailed verifying the focus, lighting, and capturing images from different angles to accommodate variations in husks. After the completion of the image collection process, we proceeded to implement quality control measures. This specific step played a crucial role in ensuring the inclusion of only high-quality images for subsequent analysis. By removing these substandard images, we have successfully preserved the integrity of the dataset and improved the reliability of subsequent analysis. A total of 2,880 husk images were initially captured. From this pool, a subset of 2,400 images was carefully selected to form the dataset proposed for this study.

### 4.2 Image preprocessing

In the beginning, a total of 2880 images were captured, featuring various species of husks. Both phones have been set to the same dimension, so all photos will seem to be the same size. After the collection of images, we conducted a manual review to remove pictures that exhibited poor quality, such as those with motion blur, excessive noise, inappropriate backgrounds, etc. Following the completion of image preprocessing, our dataset currently consists of a total of 2400 original images.

### 4.3 Image partitioning

Following the use of image preprocessing techniques, the original picture collection consists of a total of 2400 images. Next, create eight distinct directories to store the original image datasets. Subsequently, every folder underwent a renaming process, when eight distinct husk species names were assigned to each folder, along with their respective scientific names. Ultimately, the images were divided and organized into several folders based on their respective species. To train and evaluate the machine learning (ML) and deep learning (DL) models, the original images were randomly partitioned into training, validation, and test subsets. This partitioning was done at a ratio of 80:10:10 for each category.

### 4.4 Image augmentation

By adding different images through image augmentation, machine learning and deep learning-based classification models can be used in more situations and work better overall. We utilized the Keras ImageDataGenerator class to augment the quantity of images. The fill_mode property was set to the nearest to apply various picture augmentation techniques to the mentioned class. These techniques included random rotation, random noise, and horizontal flips. Before organizing images into class-based folders for researchers to access based on their requirements, image augmentation was applied to all the collected images.

### 4.5 Husk species image classification

**Fig. 6:**
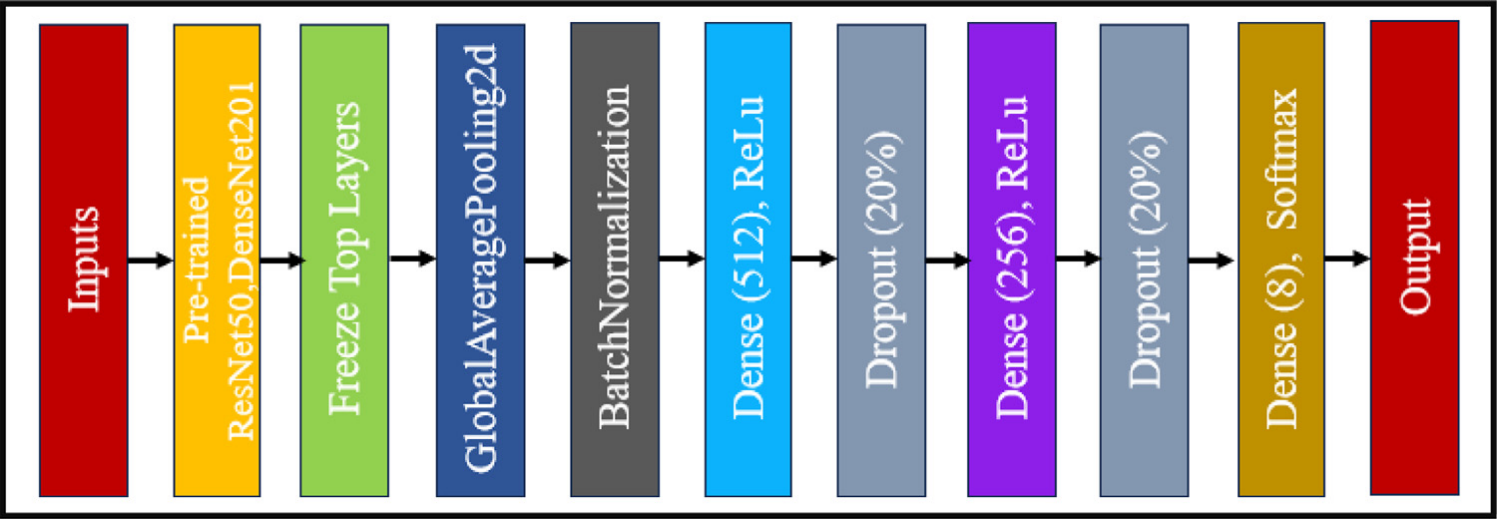
The architecture of the CNN model utilized for BDHusk evaluation.

We have used two popular and lightweight Convolutional Neural Network (CNN)-based deep learning (DL) models to assess the efficacy of the proposed “BDHusk” dataset. ResNet50 and DenseNet201 are the names of the two CNN frameworks under question. The ResNet50 model, a convolutional neural network (CNN), has made a substantial impact on the domains of computer vision and deep learning. The deep architecture of the model, consisting of 50 layers, has gained significant recognition due to its ability to effectively learn and represent intricate features within images. The ResNet50 architecture is distinguished by its implementation of a novel residual learning framework. This framework incorporates shortcut connections, which serve to facilitate the direct flow of information throughout the network. By enabling this direct flow, ResNet50 effectively reduces the vanishing gradient problem that often arises in deep neural networks. DenseNet201, an esteemed pre-trained deep learning model, derives its name from its architectural design, characterized by densely connected convolutional networks comprising a staggering 201 layers. The primary application of image classification lies in its utilization for various purposes. This neural network, known as DenseNet, is classified within the convolutional neural network family. Both pre-trained models can be easily integrated into our dataset classification problem due to their availability in popular deep-learning frameworks such as TensorFlow and PyTorch. To train our model, we use our original dataset and partition it into three distinct folders. The training dataset comprises eighty percent of the data, while the validation dataset and test dataset each account for ten percent. This can be accomplished using a widely used Python library called python_splitter, which proves to be highly advantageous for dataset splitting purposes. After partitioning the dataset, each subfolder within the train, validation, and test folders contains eight subfolders representing our species classes. The images are preprocessed using the ImageDataGenerator Python package prior to model training. Each image in the collection is resized to dimensions of (224 × 224) using three channels. The reason for this is that both of our pre-trained models have been designed to accommodate an image size of 224 × 224 × 3. Both models were constructed using the pre-trained weights from ImageNet, and the top layers of both architectures were frozen, except for the final dense layer. Two fully connected networks (FCNs) with 512 and 256 neurons, respectively, were constructed after obtaining all the pre-trained features from both designs using a global average pooling operation and batch normalization. The dense layer in this model utilized the Rectified Linear Unit (ReLU) activation function, and a dropout rate of 20% was applied to the neurons prior to the output layer. The final, fully connected layer of our model is designed to accommodate the eight distinct classes in our dataset. It consists of eight neurons and utilizes the SoftMax activation function. Consequently, there will be multiple classes within the categorization. Fig. 6 displays the architectural design of the pre-trained networks utilized for evaluating the BDHusk dataset.

The tables below provide an overview of the fully connected network architecture used to assess this dataset using ResNet50 and DenseNet201 (Tables 1 and 2).

**Table 1:** Pre-trained ResNet50-FCN architecture summary.

Layer	Shape	Number of parameters
Input layer	(None, 224, 224, 3)	0
ResNet50 (Functional)	(None, 7, 7, 2048)	23587712
Global average pooling 2D	(None, 2048)	0
Batch normalization layer	(None, 2048)	8192
Dense layer (512 units)	(None, 512)	1049088
Dropout (20%)	(None, 512)	0
Dense layer (256 units)	(None, 256)	131328
Dropout (20%)	(None, 256)	0
Dense layer (8 units)	(None, 8)	2056

Total params: 24, 778, 376.

Trainable params: 1, 186, 568.

Non-trainable params: 23, 591, 808.

**Table 2:** Summary of the pre-trained DenseNet201-FCN architecture.

Layer	Shape	Number of parameters
Input layer	(None, 224, 224, 3)	0
DenseNet201 (Functional)	(None, 7, 7, 1920)	18321984
Global average pooling 2D	(None, 1920)	0
Batch normalization layer	(None, 1920)	7680
Dense layer (512 units)	(None, 512)	983552
Dropout (20%)	(None, 512)	0
Dense layer (256 units)	(None, 256)	131328
Dropout (20%)	(None, 256)	0
Dense layer (8 units)	(None, 8)	2056

Total params: 19, 446, 600.

Trainable params: 1, 120, 776.

Non-trainable params: 18, 325, 824.

**Fig. 7:**
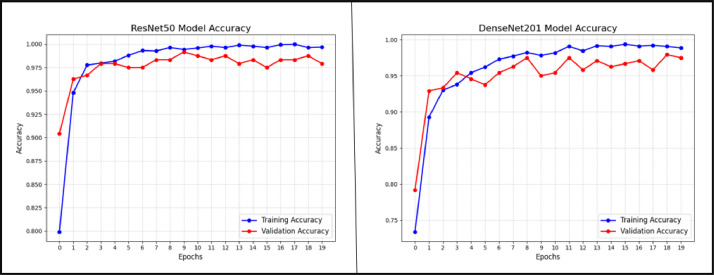
The accuracy of ResNet50 and DenseNet201 during training and validation is contrasted.

**Fig. 8:**
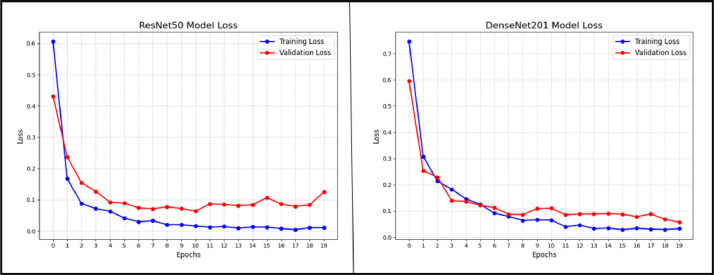
Evaluate ResNet50 and DenseNet201 in terms of their respective loss values.

During model training, an optimizer is used to try to keep the loss as small as possible. After developing and building the model, we used the Adamax optimizer and started training with a learning_rate of 0.001. Adamax is an infinity-norm variant of Adam, a first-order gradient-based optimization method. It can adjust the learning rate in response to the properties of the input, making it useful for learning time-variant processes. We then trained our model for 20 epochs after compiling it. After finishing the model training, we plotted the results to see how well the model performed throughout training and validation. In Fig. 7, a comparison is presented between the training accuracy and validation accuracy of ResNet50 and DenseNet201. The accuracy plot showcases the performance of training and validation data, with the X-axis representing accuracy and the Y-axis representing the number of epochs. In Fig. 8, we compare the loss values of the ResNet50 and DenseNet201 models over a span of 20 epochs. Training and validation loss values are shown on the X-axis, and the total number of epochs spent training both models is shown on the Y-axis in the loss plot.

**Fig. 9:**
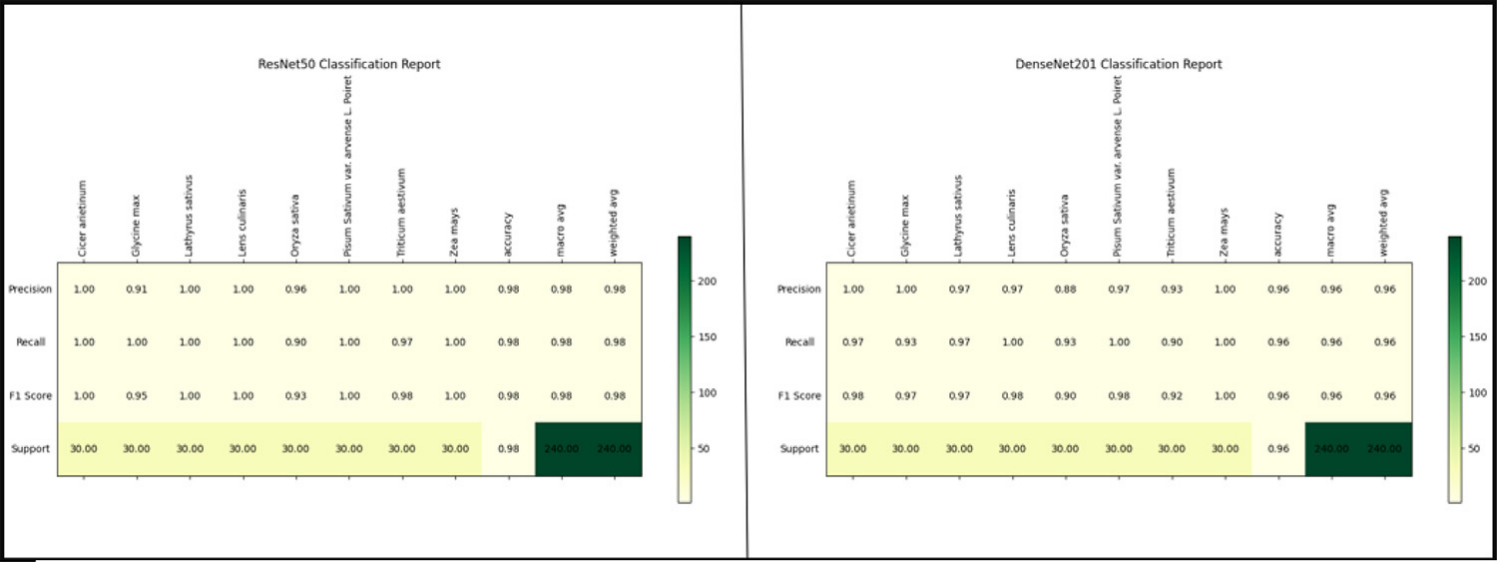
The classification report comparison between the ResNet50-FCN and DenseNet201-FCN models.

**Fig. 10:**
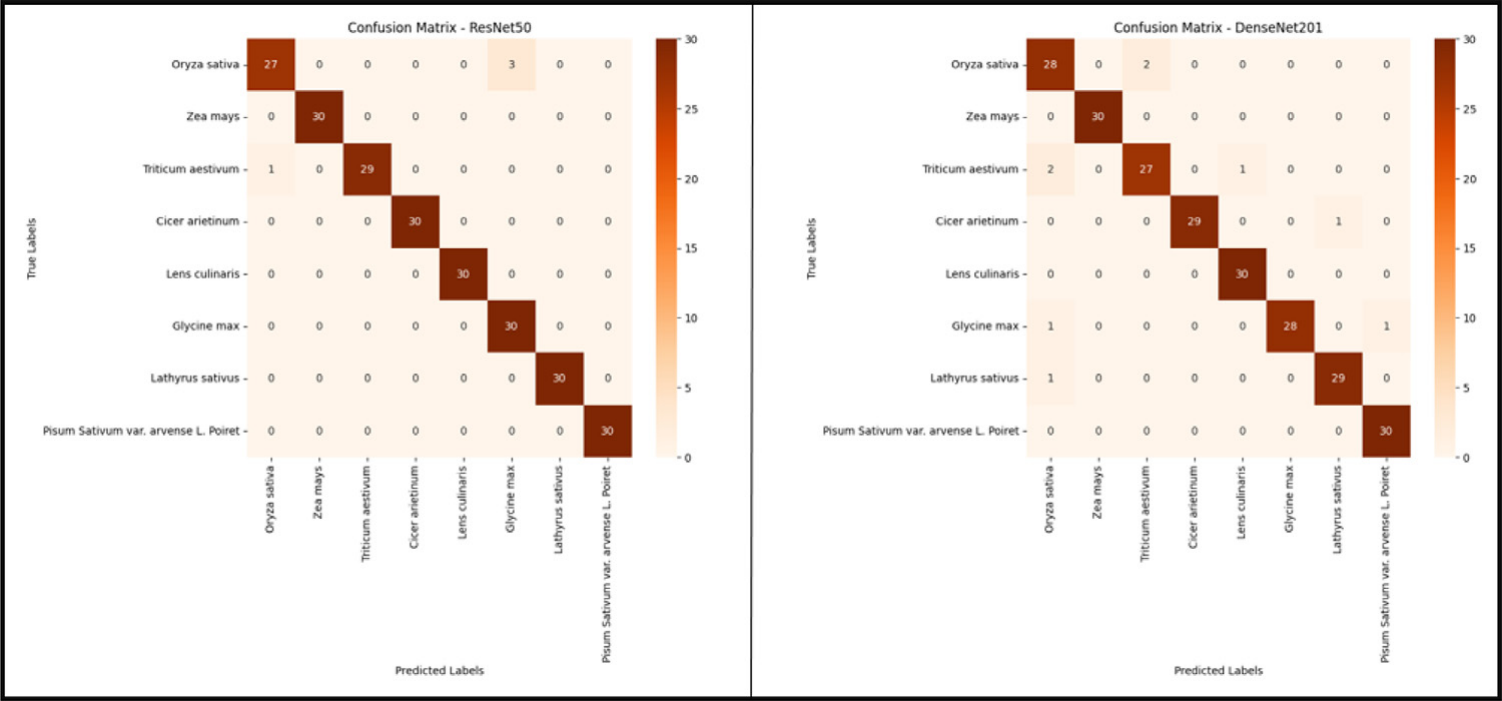
The confusion matrix for the ResNet50-FCN and DenseNet201-FCN models is presented.

The model was assessed using the test data, and a classification report and confusion matrix were generated. These reports provide information on the precision, recall, and F-1 score for each species or class within our dataset. The confusion matrix facilitates the visualization of accurate predictions and misclassifications for each class by both models. The classification report of the ResNet50-FCN and DenseNet201-FCN models is depicted in Fig. 9. The report provides a clear representation of the Precision, Recall, and F-1 score. Fig. 10 displays the confusion matrix of ResNet50-FCN and DenseNet201-FCN.

The presented results demonstrate the effectiveness of deep learning models in accurately classifying husk species using the recommended “BDHusk” dataset. During the evaluation of the pre-trained model perfor, it was observed that although the training accuracy of both models was the same, there was a discrepancy in their validation accuracy. Over the course of the last 10 epochs, the validation accuracy of the DenseNet201-FCN model has demonstrated a consistent level of performance, closely aligned with the training accuracy. The validation accuracy of ResNet50 has exhibited considerable variability throughout the training process. However, in terms of class predictions using test data, ResNet50-FCN demonstrates slightly superior performance compared to DenseNet201-FCN. The ResNet50 model accurately classified Cicer arietinum, Lathyrus sativus, Lens culinaraestivum justvum var. arvense L. Poiret, and Zea mays with 100% accuracy. However, it misclassified one data point of Triticum aestivum as Oryza sativa and misclassified three data points of Oryza sativa as Glycine max. The DenseNet201 model achieved accurate classification of Zea mays with a 100% appropriately and misclassified. In DenseNet201 model, Oryza sativa has two incorrect data as Triticum aestivum and Triticum aestivum misclassified three times, two times as Oryza sativa and one time as Lens culinaris. The performance overview of the ResNet50-FCN model and the DenseNet201-FCN model can be seen in Table 3.

**Table 3:** Performance of the pre-trained ResNet50-FCN and DenseNet201-FCN For *BDHusk* dataset.

Model	Train accuracy (%)	Validation Accuracy (%)	Test accuracy (%)
Pre-trained ResNet50-FCN (224 × 224 × 3)	99.95%	97.50%	96.25%
Pre-trained DenseNet201-FCN (224 × 224 × 3)	99.95%	97.50%	96.25%

## Limitations

In the regions from which we have gathered data, it has been observed that a variety of husks are utilized as cattle feed. However, it is worth noting that among the numerous options available, our findings indicate that these eight specific types of husks are mostly preferred, while other variations are hardly seen above these regions. That is why only these eight types of husks are used in our dataset.

## Ethics Statement

The ethical concerns pertaining to the dataset on Husk involve a dedication to conscientious data collecting and usage. The production of this collection is guided by principles that prioritize justice, openness, and environmental respect. Considerable effort was made to ensure that the collecting procedure was conducted in a manner that did not cause harm to any live animals or ecosystems. It is imperative to acknowledge that all husk images were obtained with explicit consent from owners. The authors have thoroughly reviewed and adhered to the ethical guidelines for publishing in Data in Brief. They have ensured that their research does not involve human subjects, animal experimentation, or the use of data obtained from social media platforms.

## CRediT authorship contribution statement

**Ifteasam Islam Jahin:** Data curation, Formal analysis, Software, Conceptualization, Methodology, Investigation, Writing – original draft, Visualization. **Munni Khatun:** Data curation, Formal analysis, Writing – original draft, Resources, Conceptualization, Methodology, Software, Investigation. **Md. Tarequl Islam:** Conceptualization, Formal analysis, Project administration, Supervision, Writing – review & editing, Methodology, Resources. **Md. Wahidur Rahman:** Conceptualization, Formal analysis, Supervision, Project administration, Writing – review & editing, Software, Validation. **Ishrat Zahan Raka:** Formal analysis, Conceptualization, Project administration, Supervision, Writing – review & editing.

## Declaration of Competing Interest

The authors hereby confirm that they currently possess no existing financial or interpersonal conflicts that could have potentially influenced the outcomes of this study.

## Data Availability

BDHusk: A Comprehensive Dataset of Different Husk Species Images as a Component of Cattle Feed from Different Regions of Bangladesh. (Original data) (Mendeley Data).
